# Phosphorylation of Human Choline Kinase Beta by Protein Kinase A: Its Impact on Activity and Inhibition

**DOI:** 10.1371/journal.pone.0154702

**Published:** 2016-05-05

**Authors:** Ching Ching Chang, Ling Ling Few, Manfred Konrad, Wei Cun See Too

**Affiliations:** 1 School of Health Sciences, Health Campus, Universiti Sains Malaysia, 16150 Kubang Kerian, Kelantan, Malaysia; 2 Enzyme Biochemistry Group, Max Planck Institute for Biophysical Chemistry, 37077, Goettingen, Germany; University of Padova, ITALY

## Abstract

Choline kinase beta (CKβ) is one of the CK isozymes involved in the biosynthesis of phosphatidylcholine. CKβ is important for normal mitochondrial function and muscle development as the lack of the *ckβ* gene in human and mice results in the development of muscular dystrophy. In contrast, CKα is implicated in tumorigenesis and has been extensively studied as an anticancer target. Phosphorylation of human CKα was found to regulate the enzyme’s activity and its subcellular location. This study provides evidence for CKβ phosphorylation by protein kinase A (PKA). *In vitro* phosphorylation of CKβ by PKA was first detected by phosphoprotein staining, as well as by in-gel kinase assays. The phosphorylating kinase was identified as PKA by Western blotting. CKβ phosphorylation by MCF-7 cell lysate was inhibited by a PKA-specific inhibitor peptide, and the intracellular phosphorylation of CKβ was shown to be regulated by the level of cyclic adenosine monophosphate (cAMP), a PKA activator. Phosphorylation sites were located on CKβ residues serine-39 and serine-40 as determined by mass spectrometry and site-directed mutagenesis. Phosphorylation increased the catalytic efficiencies for the substrates choline and ATP about 2-fold, without affecting ethanolamine phosphorylation, and the S39D/S40D CKβ phosphorylation mimic behaved kinetically very similar. Remarkably, phosphorylation drastically increased the sensitivity of CKβ to hemicholinium-3 (HC-3) inhibition by about 30-fold. These findings suggest that CKβ, in concert with CKα, and depending on its phosphorylation status, might play a critical role as a druggable target in carcinogenesis.

## Introduction

Choline kinase (CK) phosphorylates choline in the cytidine diphosphate (CDP)-choline pathway for the biosynthesis of phosphatidylcholine (PC), the most abundant class of phospholipids in eukaryotic membranes [1]. In mammals, three CK isozymes exist, known as CKα1, CKα2, and CKβ. CKα1 and α2 are derived from an alternatively spliced *ckα* gene (CHKA gene ID: 1119) [2], whereas CKβ is the product of the *ckβ* gene (CHKB gene ID: 1120) [3, 4]. CKβ has similar enzymatic activity as CKα, but with a lower catalytic efficiency [5], and has a distinct physiological role for normal mitochondrial function [6]. In both humans and mice with the CKβ gene mutated or deleted, mitochondrial dysfunction and degeneration were observed in muscle cells [7–9]. *ckβ* knockout mice developed forelimb bone deformity and hindlimb muscular dystrophy [6]. Individuals carrying *ckβ* gene deletion showed congenital muscular dystrophy with early onset muscle wasting and mental retardation [9]. Histologically, mitochondria in the muscle of *ckβ* knockout mice were extremely enlarged with peripheral location of the nuclei, which indicated mitochondrial myopathy and absence of mitochondrial proliferation [8]. Tissue biopsy from knockout mice showed reduced levels of total PC [8]. In such tissue, no compensation by the CKα isozyme for the loss of CKβ was detected. Similarly, *ckβ* did not counterbalance defects observed in *ckα* knockout mice that died in early embryonic development [10]. Recently, CKβ was reported to play an important role in maintaining bone homeostasis, notably via regulation of osteoclast and osteoblast functions, and bone deformation in *ckβ* knockout mice being specific to the radius and ulna during late embryonic stage [11, 12].

Regulation of the CDP-choline pathway is important for normal phospholipid metabolism and cell growth: CK catalyzes the first committed step and thus plays a critical role in the regulation of this pathway especially at high choline concentration [13]. Dysregulation of CK is associated with tumorigenic transformation [14–17]. Thus, in cancerous cells, the catalytic activity of CK and the concentrations of choline metabolites were shown to be elevated [18, 19], and therefore CK has been regarded as a cancer marker and became a potential target for antitumor therapy [15, 16]. Cellular and biochemical analyses identified CKα, but not CKβ, as the isozyme that is responsible for tumorigenic transformation [20]. Although CKβ itself is not directly associated with cell transformation and tumor development, the balance between CKβ and CKα is important for cell cycle regulation [21]. In addition, a differential role of CKα and CKβ in lipid metabolism was reported whereby CKβ was shown to exhibit ethanolamine kinase activity and contributes to distinct biochemical pathways under *in vivo* conditions [20].

In order to reveal potential molecular mechanisms involved in regulating CK activity, we studied the role of phosphorylation as one of the major types of post-translational modifications regulating protein function. Phosphorylation is rapid and reversible, it adds one or more negatively charged groups onto amino acid side chains of a protein, altering the local charge of the protein [22]. In mammals, about one third of the cellular proteins are phosphorylated [23]. Phosphorylation of human CKα and yeast CK has been reported to enhance the catalytic activity of the enzymes and elevate the biosynthesis of PC, phosphorylation of CKα being c-Src dependent [24], whereas yeast CK was directly phosphorylated by protein kinase A (PKA) and protein kinase C (PKC) [25–27]. Phosphorylation of CKα induced the interaction of CKα with the epidermal growth factor receptor (EGFR), and upon complex formation, CKα was translocated from the cytosol to the plasma membrane of the cell [24]. Similarly, CTP-phosphocholine cytidylyltransferase (CCT), the second enzyme in the CDP-choline pathway, is also regulated by phosphorylation: It is phosphorylated at multiple residues by different protein kinases such as PKA [28], mitogen-activated protein kinase (MAPK) [29], c-Jun N-terminal kinases (JNK) [30], and calmodulin kinase I (CaMKI) [31], thereby regulating its subcellular location and activity [32, 33].

In contrast to CKα, phosphorylation and regulation of CKβ have not been reported. When we analyzed CKβ for potential phosphorylation sites using NetPhosK 1.0 [34] and ScanProsite [35], CKβ was predicted to be phosphorylated by several protein kinases, two of them being PKA and PKC that had previously been reported to phosphorylate yeast CK [25, 27]. In this study, we used in-gel kinase and *in vitro* phosphorylation assays to determine the protein kinase responsible for the phosphorylation of CKβ. We identified the phosphorylation sites and studied consequences of PKA phosphorylation for CKβ catalytic activity and sensitivity to inhibition by the small-molecule drug hemicholinium-3 (HC-3).

## Results

### Identification of CKβ-phosphorylating protein kinase

**Fig 1:**
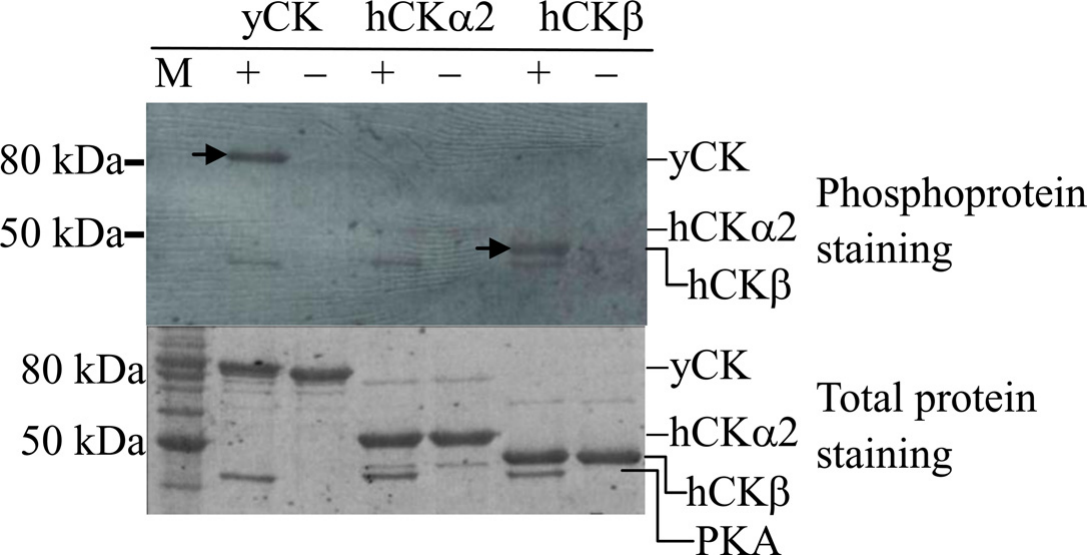
Protein kinase A phosphorylation of recombinantly produced and purified yeast (yCK) and human (hCK) choline kinases. yCK, hCKα2, and hCKβ were treated (+) with 80 U of PKA catalytic subunit. Unphosphorylated samples are indicated by “−”. Arrows indicate positive phosphoprotein staining for yCK and hCKβ. M: BenchMark™ Protein Ladder. Ten micrograms of the phosphorylated proteins were analyzed by 10% SDS-PAGE. Phosphorylated and total proteins were stained with Pro-Q Diamond phosphoprotein stain and SYPRO^®^ Ruby stain, respectively.

PKA is one of the protein kinases responsible for the phosphorylation of yeast choline kinase [36]. Our computational prediction using NetPhosK 1.0 [34] and ScanProsite [35] revealed the possibility of human CK phosphorylation by PKA. Therefore, *in vitro* PKA phosphorylation of recombinantly produced human CK isozymes was probed. Yeast CK was included as a positive control. Fig 1 shows that CKβ, but not CKα, was phosphorylated by PKA as indicated by phosphoprotein signal intensity similar to that of yeast CK.

**Fig 2:**
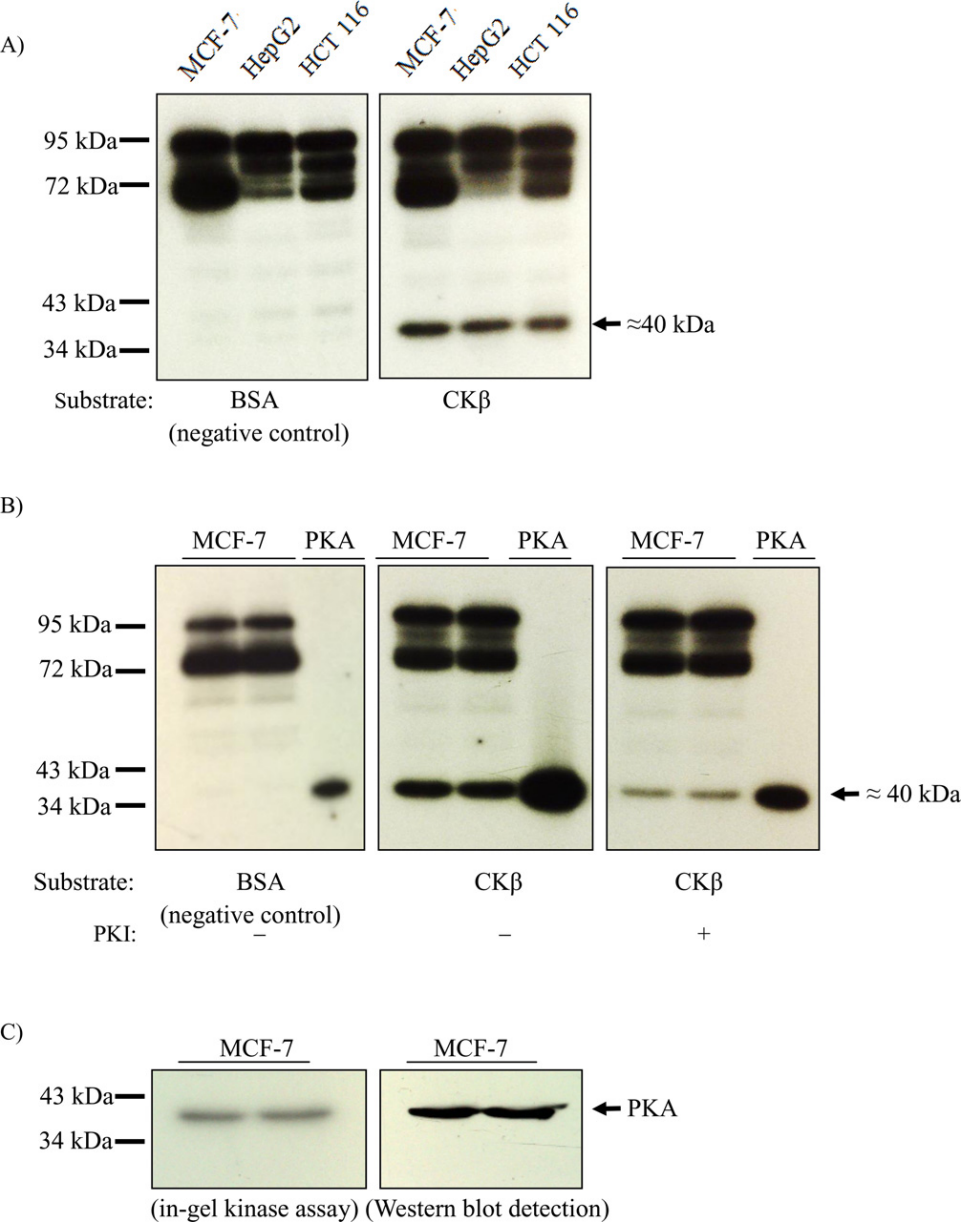
Identification of PKA as the protein kinase responsible for the phosphorylation of CKβ. A) In-gel kinase assay of CKβ with MCF-7, HepG2, and HCT-116 as the sources of protein kinase PKA. Two milligrams of CKβ were used as phosphorylation substrate in the gel, and 60 μg of cell lysate were applied in each lane. After electrophoresis, the gel was washed, incubated in denaturation and renaturation buffers as described in the experimental procedures, followed by the kinase reaction by incubating the gel with 50 μM of radiolabelled ATP for 4 hours. The reaction was terminated by incubating the gel in stop buffer. CKβ phosphorylation was detected by autoradiography. B) Effect of specific PKA peptide inhibitor (PKI) on the activity of the 40 kDa protein kinase. The in-gel kinase assay was run as described above with 15 μg/mL of PKI included in the kinase buffer. MCF-7 cell lysates were run in duplicate lanes. C) Western blot detection of the 40 kDa protein kinase by a specific anti-PKA antibody. A replicated gel of the in-gel kinase assay before the kinase reaction step was blotted onto a nitrocellulose membrane followed by immunodetection with 1:5,000 dilution of anti-PKA catalytic subunit polyclonal antibody.

An in-gel kinase assay was performed to determine whether CKβ is phosphorylated by PKA in mammalian cell lysates. In this technique, CKβ protein or BSA (negative control) was co-polymerized within the gel matrix used to separate protein kinases in cell lysates based on molecular size. Bands detected between 72 to 95 kDa with BSA and CKβ as substrates in Fig 2A and 2B are not specific to CKβ and were excluded from further analysis. As shown in Fig 2A, CKβ was specifically phosphorylated by an approximately 40 kDa protein kinase from all three cell lines (MCF-7, HepG2, and HCT-116) tested. The apparent molecular weight of the CKβ-phosphorylating protein kinase corresponds to the size of the PKA catalytic subunit. A specific PKA inhibitor peptide (PKI) was used to verify the identity of the ≈40 kDa protein kinase. Only MCF-7 cell lysate was used in this experiment because the level of in-gel kinase phosphorylation was found to be similar for all three cell lines. Fig 2B shows that the phosphorylation levels attained by the ≈40 kDa-protein kinase and the positive control (commercial recombinant PKA catalytic subunit) were reduced by the addition of PKI in the in-gel kinase assay. This result supports the notion that the ≈40 kDa protein kinase is the PKA catalytic subunit. On the negative control gel, autophosphorylation of recombinant PKA, which is the mechanism for its activation [37], was detected. However, autophosphorylation of the much less concentrated native PKA catalytic subunit in the cell lysate was not observed. A specific PKA antibody was used to confirm the identity of the ≈40 kDa protein kinase: The band detected by immunoblotting corresponds to the band from the in-gel kinase assay of the same gel (Fig 2C). Based on these results, we conclude that PKA is the protein kinase responsible for the phosphorylation of CKβ.

### *In vitro* phosphorylation of CKβ by MCF-7 cell lysate and PKA

**Fig 3:**
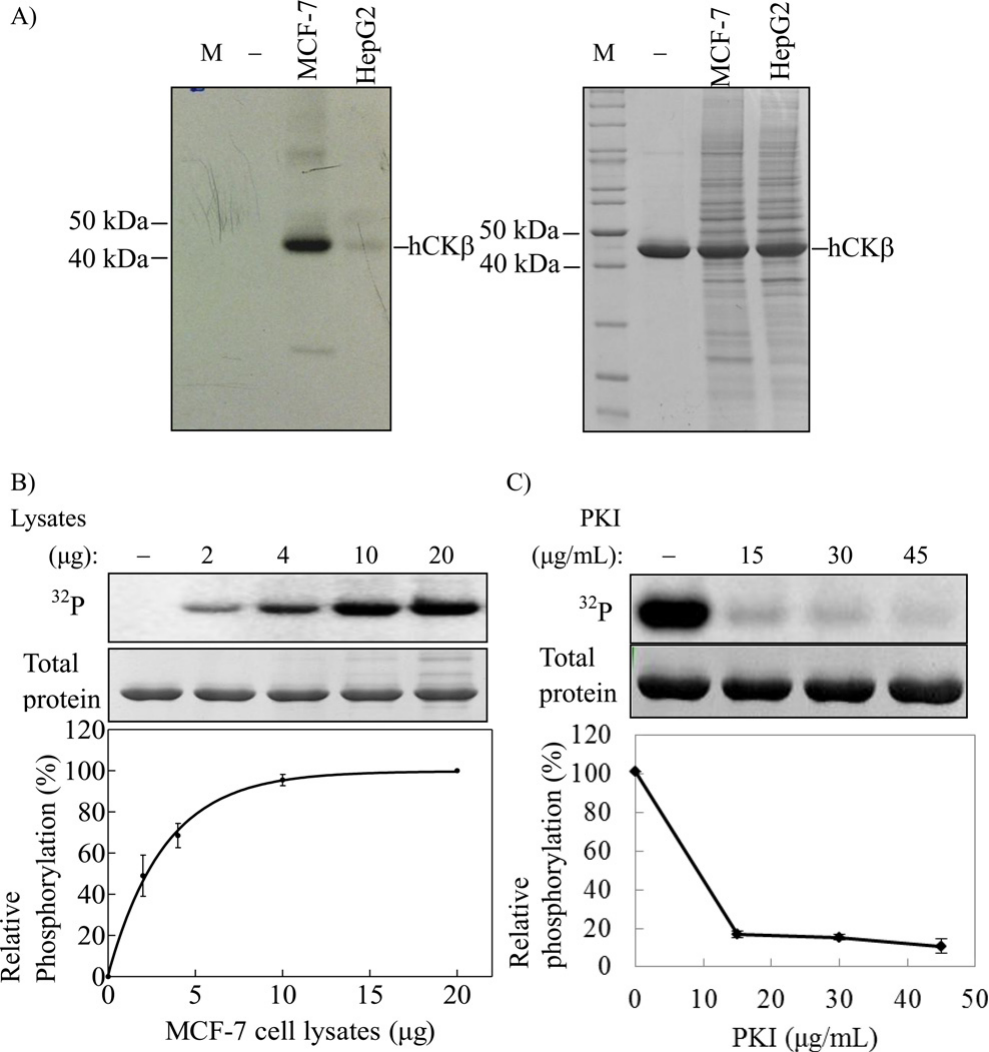
Phosphorylation of purified CKβ by PKA in MCF-7 cell lysate. A) *In vitro* phosphorylation of CKβ by protein kinase in 20 μg each of MCF-7 and HepG2 cell lysates. Twenty micrograms of CKβ were *in vitro* phosphorylated for 1 hour at 30°C as described in the experimental procedures. Five micrograms of the phosphorylated proteins were analyzed by 10% SDS-PAGE. The right panel shows Coomassie blue total protein staining of the same gel. Phosphorylated CKβ was detected by autoradiography. B) The phosphorylation level of CKβ is dependent on the amount of MCF-7 cell lysate. C) The phosphorylation of CKβ is inhibited by PKI: Here, 20 μg of MCF-7 cell lysate were used to phosphorylate CKβ. The relative amounts of phosphate incorporated were quantified by using ImageJ 1.42. M: PageRuler™ unstained protein ladder.

To unravel the potential physiological protein kinase, total MCF-7 and HepG2 cell lysates were also used to phosphorylate the purified recombinant CKβ. In comparison with in-gel kinase assay, the use of cell lysates offers the possibility to test for native protein kinases without denaturation and renaturation steps of the kinase in cell lysate. Fig 3A (left panel) shows that both MCF-7 and HepG2 cell lysates were able to phosphorylate CKβ, with a stronger phosphorylation signal originating from the same amount of MCF-7 lysate, indicating that more active protein kinase was present in MCF-7 than in HepG2 cell lysate preparations. The phosphorylation level of CKβ was dependent on the amount of MCF-7 cell lysate used (Fig 3B, left panel). The relative CKβ phosphorylation level was about 80% using 6 μg of MCF-7 cell lysate and reached a plateau at about 10 μg cell lysate per reaction. A protein kinase inhibition assay in the presence of PKI was performed to show that PKA was the major protein kinase in MCF-7 cell lysate that phosphorylated CKβ. As shown in Fig 3C, the relative phosphorylation of CKβ by MCF-7 cell lysate dropped to less than 20% with 15 μg/mL of PKI, and to less than 10% in the presence of 45 μg/mL of PKI. These data provide further supporting evidence for PKA being the primary protein kinase that phosphorylates CKβ.

**Fig 4:**
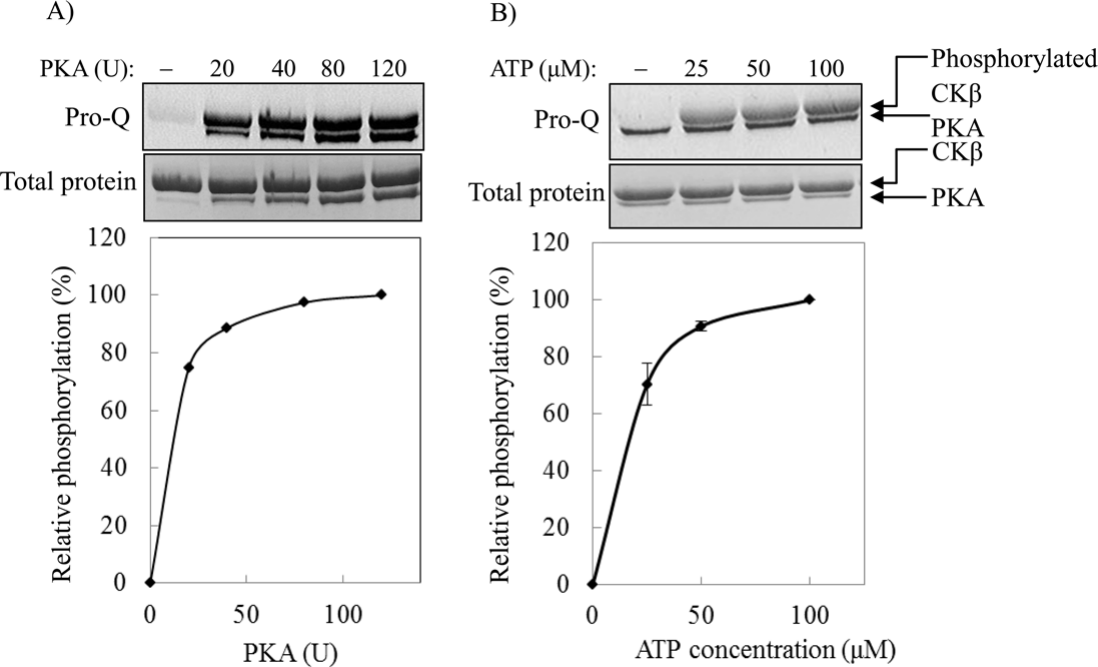
Effect of PKA and ATP concentrations on the level of CKβ phosphorylation. The phosphorylation level of His-tagged CKβ by commercial recombinant PKA catalytic subunit was detected with Pro-Q Diamond phosphoprotein gel stain (upper panel). Middle panel shows the total protein staining with Coomassie blue. The signals of phosphorylated CKβ were quantitated with Image J 1.42 and plotted as relative phosphorylation versus A) units of PKA (1 mM ATP was used), and B) concentration of ATP (80 units of PKA were used). Each bar represents the standard error of mean from two independent experiments.

To corroborate this view, we carried out experiments using commercial recombinant PKA catalytic subunit to investigate the effect of PKA and ATP concentrations on the phosphorylation of CKβ. Fig 4A shows that the relative level of phosphorylated CKβ increased with the higher activity of PKA in the reaction. The relative level of phosphorylated CKβ was also dependent on the ATP concentration in the reaction (Fig 4B). Thus, these results confirm that CKβ is a substrate for PKA. The relative phosphorylation level of CKβ reached saturation at about 80 U of PKA and 100 μM of ATP. These (or higher) amounts of PKA and ATP were used in the following experiments to ensure maximum PKA phosphorylation of CKβ.

### Identification of PKA phosphorylation sites in CKβ

**Fig 5:**
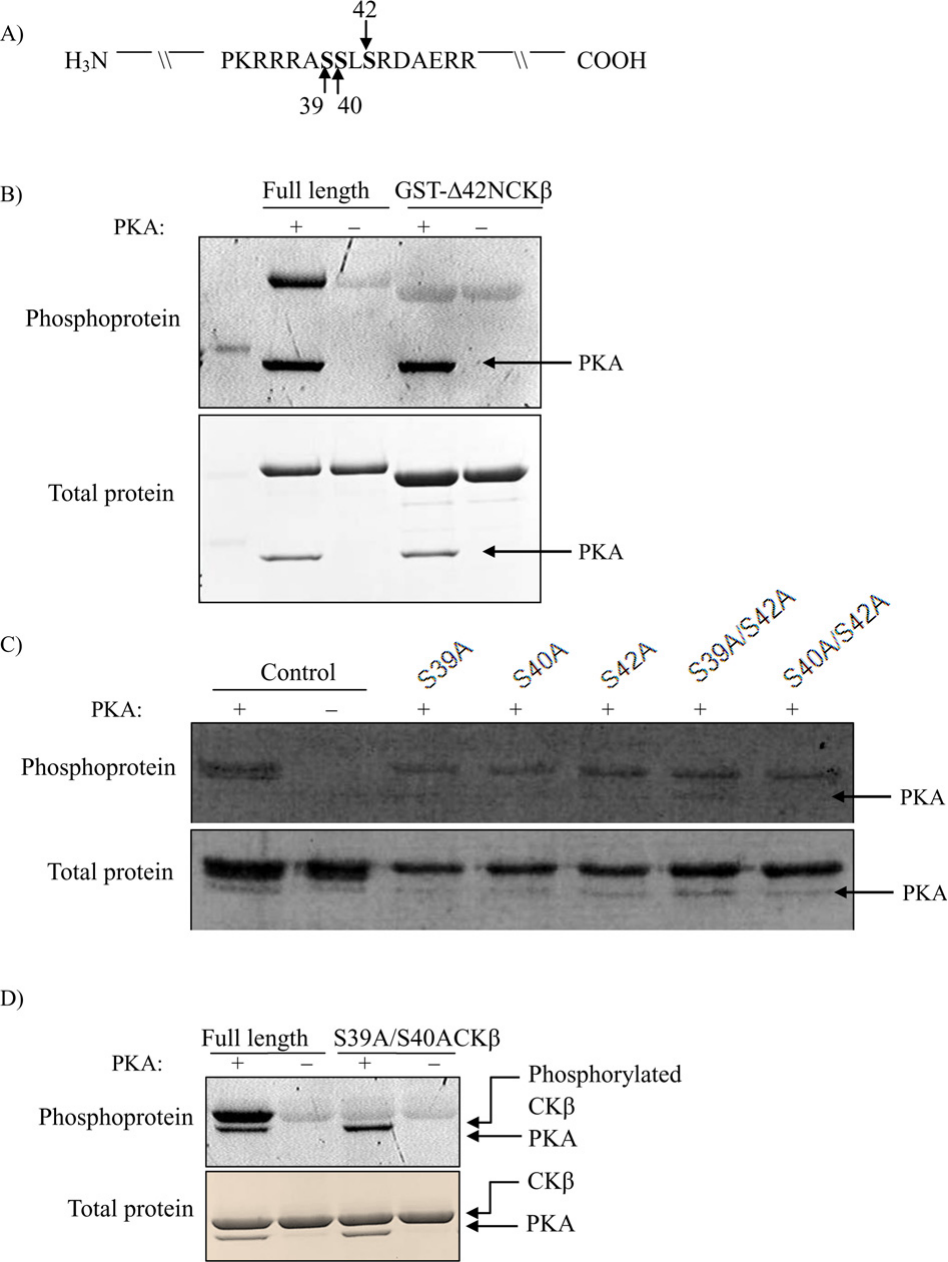
Identification of Ser-39 and Ser-40 in hCKβ as the major sites of PKA phosphorylation. A) Schematic representation of potential sites of PKA phosphorylation in hCKβ as determined by mass spectrometry. *In vitro* phosphorylation of B) GST-hCKβ and GST-∆42NhCKβ recombinant fusion proteins, C) hCKβ, S39AhCKβ, S40AhCKβ, S42AhCKβ, S39A/S42AhCKβ, S40A/S42AhCKβ, and D) S39A/S40AhCKβ mutant proteins. The pictures are representative of at least two independent experiments. All CKβ mutant proteins were *in vitro* phosphorylated with 80 U PKA as described in the experimental procedures. Five micrograms of phosphorylated CKβ were loaded in each lane, and phosphorylation was detected with Pro-Q Diamond phosphoprotein stain. Total protein was stained with either Coomassie blue (B & D) or SYPRO^®^ Ruby stain (C).

PKA phosphorylation sites in CKβ were determined by mass spectrometry, and their functional relevance was studied by mutagenesis. The mass spectrometry identification of phosphorylation sites was done using purified and *in vitro* phosphorylated CKβ. Mass spectrometry detected the occurrence of phosphorylation on serine residues 39, 40 and 42 at the highest frequency. Our NetPhosK 1.0 [34] phosphorylation prediction scores for these serine residues were 0.995, 0.996 and 0.977, respectively. Mascot Search analysis always showed serine and threonine phosphorylation by the neutral loss of phosphate (either H_3_PO_4_ of 98 Da or HPO_3_ of 80 Da), while tyrosine fragment ions stayed intact (additional mass of 98 Da or 80 Da). Fifteen peptides were found to be phosphorylated, the ion score ranging from 4 to 36. Out of these 15 fragments, 13 were identified as peptides covering the stretch of amino acids from R36 to R43 (RRASSLSR) harboring three serine residues. Simultaneous phosphorylation of both S39 and S40 occurred seven times, whereas phosphorylation of other sites, either individually (S40; S42) or in combination (S39 and S42; S40 and S42; S39 and S40 and S42) occurred only once; S39 phosphorylation alone was not found (Table 1). The full results of mass spectrometry analysis of CKβ phosphorylation are given in S1 Fig. Fig 5A depicts amino acids surrounding the phosphorylated serines: This region contains the PKA consensus sequence RRXS/TY, where X is any residue while Y is a hydrophobic residue [38]. To demonstrate that PKA phosphorylation of CKβ was confined to the N-terminal part of the protein (up to serine 42), a GFP-tagged 42-residue N-terminally truncated mutant of CKβ was produced and tested for *in vitro* phosphorylation by the PKA catalytic subunit. Fig 5B shows that the phosphorylation level of Δ42NCKβ was similar to the negative control (without PKA in the reaction), indicating that PKA phosphorylation only occurred within the first 42 amino acids of the CKβ protein. The PKA phosphorylation sites identified by mass spectrometry were further verified by serine-to-alanine mutations, either individually or in combination. The His-tagged CKβ mutants were *in vitro* phosphorylated with PKA catalytic subunit. Results in Fig 5C show that phosphorylation was still detectable in all cases of individual mutation of the three serines. This indicates that at least two of the serine residues were phosphorylated by PKA. Phosphorylation was also detectable with S39A/S42A and S40A/S42A double mutants (Fig 5C), but not with the S39A/S40A double mutant (Fig 5D). Thus, this mutational screen points to the tandem serines 39 and 40 in CKβ as the target sites for PKA phosphorylation. Since the S39A/S40A CKβ mutant was not phosphorylated by PKA, it was used as the phosphorylation-negative enzyme species in subsequent experiments.

**Table 1:** Summary of the MASCOT search analysis on the ^36^RRASSLSR^43^ peptide stretch of CKβ.

Serine residues	Ion score	Frequency of occurrences
39	−	0
40	15	1
42	15	1
39 and 40	7 − 27	7
39 and 42	4	1
40 and 42	0;10	2
39, 40 and 42	5	1
Total	−	13

### Intracellular phosphorylation of CKβ

**Fig 6:**
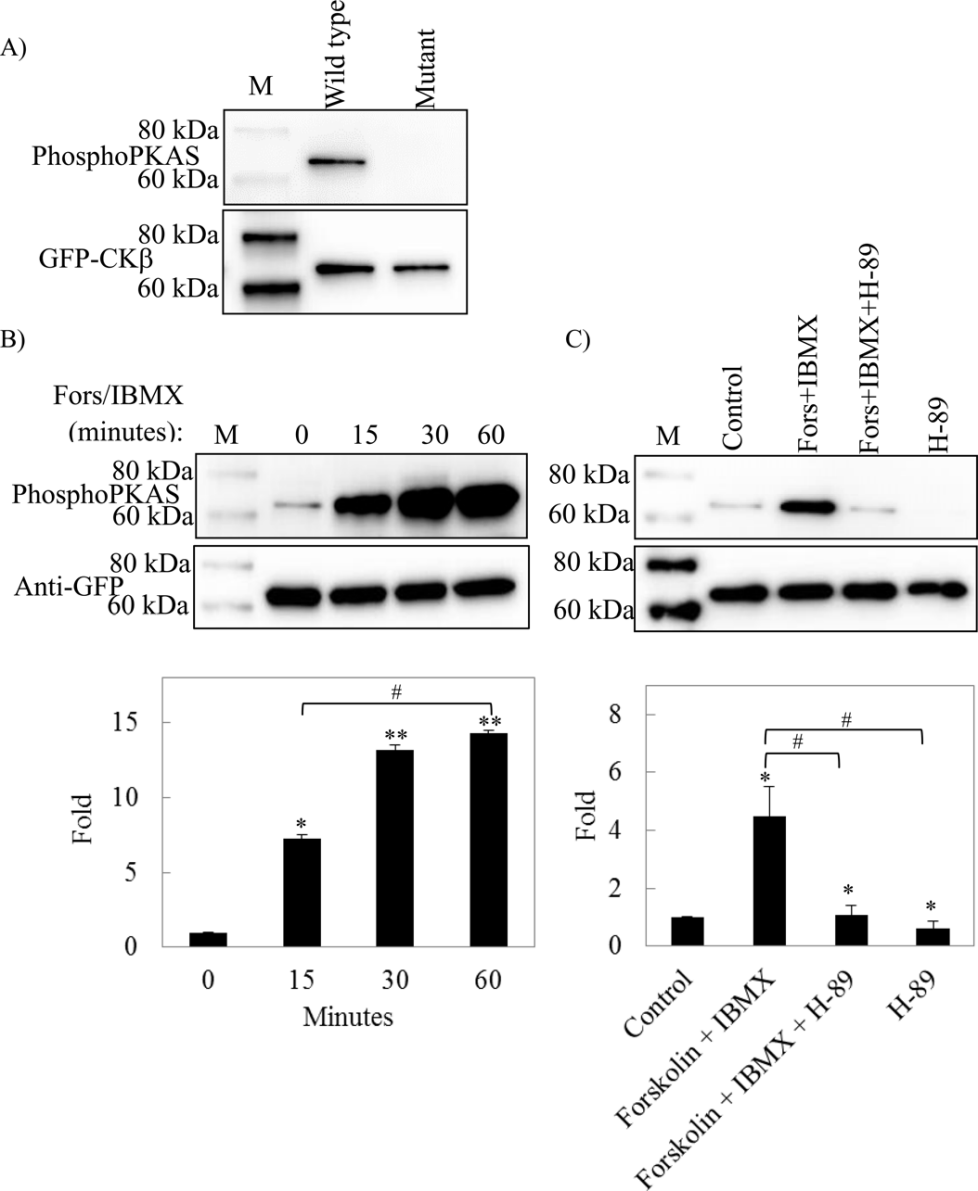
Effect of forskolin, IBMX, and H-89 treatment on the intracellular phosphorylation of GFP-CKβ. A) Phosphorylation state of the GFP-tagged wild type and S39A/S40ACKβ phosphorylation-negative mutant. B) Time-dependent effect of forskolin (fors) and IBMX treatment on the level of CKβ phosphorylation. C) Effect of H-89 treatment on the level of CKβ phosphorylation. In all experiments, the phosphorylation level of the immunoprecipitated CKβ was monitored by PhosphoPKAS antibody, and anti-GFP antibody detection was subsequently performed as the loading control. The intensities of respective bands were quantitated by Image J 1.42 software and plotted as the phosphorylation level relative to the control. Each bar represents the standard error of the mean (SEM) from two independent experiments. Statistical analysis was performed using one-way ANOVA and the Tukey HSD *post-hoc* test (*p < 0.05 vs control; **p < 0.01 vs control, #*p* < 0.05, significant between treatment group). M: Supersignal^®^ molecular weight protein ladder.

Intracellular phosphorylation of CKβ was examined by detecting the phosphorylation of GFP antibody immunoprecipitated GFP-tagged wild type and phosphorylation-negative mutant CKβ variants expressed in HEK293 cells. Based on the results shown in Fig 6A, wild type CKβ was detected as a phosphoprotein by a specific phosphoPKAS antibody. This antibody recognizes the phosphorylated serine or threonine residues on the PKA consensus phosphorylation sequence RRxS/T (RRASS in CKβ). In contrast, the phosphoPKAS antibody detected no phosphorylated proteins in immunoprecipitates of the phosphorylation-negative mutant, demonstrating that the S39A/S40A CKβ mutant did not serve as substrate for PKA phosphorylation, and wild type CKβ was phosphorylated by PKA in HEK293 cells. In addition, the specificity of the phosphoPKAS antibody in detecting intracellular PKA phosphorylation of CKβ was confirmed by these results.

### The phosphorylation level of CKβ is regulated by the level of cAMP

Activity of PKA, also known as cAMP-dependent protein kinase, is regulated by intracellular cAMP. The level of cAMP can be increased by treating the cells with forskolin and IBMX: Forskolin raises the intracellular level of cAMP by activation of adenylyl cyclase that generates cAMP from ATP, and IBMX maintains the level of cAMP by preventing its degradation by phosphodiesterase. In this experiment, H-89, a PKA inhibitor, was also used: H-89 blocks the activity of PKA through competitive binding to its ATP site on the catalytic subunit. These small effector molecules were used in this study to substantiate the intracellular phosphorylation of CKβ by PKA.

Fig 6B shows that the level of CKβ phosphorylation in HEK293 cells was raised by forskolin and IBMX treatment: 15 min after the treatment, the phosphorylation level of immunoprecipitated GFP-tagged CKβ increased significantly by 7.5-fold compared to non-treated cells, and it increased further by 13-fold as compared to control 30 min after treatment with the PKA activator. No significant increment of CKβ phosphorylation occurred between 30 min and 60 min post treatment, suggesting that PKA phosphorylation of CKβ reached the saturation level. Cells pre-treated with H-89 suppressed the forskolin and IBMX-induced phosphorylation of CKβ (Fig 6C). The level of CKβ phosphorylation in cells pretreated with H-89, but without subsequent exposure to forskolin and IBMX, was decreased to a level that was lower than that of the control (Fig 6C). Thus, these results showed that CKβ phosphorylation is regulated by the level of intracellular cAMP which modulates PKA activity.

### PKA phosphorylation alters the biochemical properties of CKβ

The effect of PKA phosphorylation on the biochemical properties of CKβ was investigated by comparing the catalytic activities of *in vitro* phosphorylated and unphosphorylated CKβ, as well as of a phosphorylation mimic mutant in which the serines 39 and 40 were substituted with aspartates. Table 2 shows the kinetic parameters determined for bacterially produced and purified wild type and mutant phosphorylated and unphosphorylated CKβs. The Michaelis-Menten plots for the determination of all the parameters listed in Table 2 are provided as S2, S3, and S4 Figs. PKA phosphorylation of wild type CKβ increased the maximum rate, V_max_, for choline, ethanolamine, and ATP by 1.6-, 2-, and 1.4-fold, respectively, as compared to the unphosphorylated enzyme. In terms of apparent substrate affinity, PKA phosphorylation lowered the value of the Michaelis constant, K_m_, when choline or ATP was used as substrate. In contrast, the K_m_ value for ethanolamine was increased upon PKA phosphorylation. As a result, PKA phosphorylation improved the catalytic efficiency (*k*_cat_/K_m_) of CKβ for ATP-dependent choline phosphorylation, but decreased the catalytic efficiency of CKβ with ethanolamine as substrate. Moreover, PKA phosphorylation changed the substrate preference of CKβ from ethanolamine to choline. As indicated by the changes in K_m_ values for choline and ethanolamine, unphosphorylated CKβ had higher affinity for ethanolamine than for choline, whereas phosphorylated CKβ preferred choline rather than ethanolamine as substrate. The kinetic parameters obtained with different substrates of the CKβ phosphorylation mimic S39D/S40D mutant were remarkably similar to those of the PKA-phosphorylated CKβ enzyme. These results provided evidence that the replacement of phosphorylated serines in CKβ with aspartates successfully mimicked the effect of PKA-induced phosphorylation of CKβ.

**Table 2:** Kinetic parameters of unphosphorylated CKβ, phosphorylated CKβ, and phosphorylation-mimic mutant of CKβ. The values (±SEM) of V_max_ and K_m_ were obtained from triplicate measurements. All activities were determined by the standard PK-LDH-dependent coupled-enzyme assays as described in the experimental procedures. For the ATP data, the phosphoryl acceptor substrate was choline at 4 mM concentration.

Substrates		Unphosphorylated CKβ	PhosphorylatedCKβ	S39D/S40D CKβ
	V_max_ (U/mg)	40.14±1.26	64.27±2.39	60.42±2.14
	K_m_(mM)	0.62±0.07	0.42±0.06	0.46±0.06
**Choline**	*k*_cat_ (s^-1^)	30.10	48.25	45.31
	*k*_cat_/K_m_ (mM^-1^s^-1^)	48.54	114.88	98.50
	V_max_ (U/mg)	14.28±0.21	28.69±0.28	28.39±0.40
	K_m_(mM)	0.26±0.01	0.55±0.01	0.57±0.02
**Ethanolamine**	*k*_cat_ (s^-1^)	10.71	21.53	21.29
	*k*_cat_/K_m_ (mM^-1^s^-1^)	41.19	39.14	37.35
	V_max_ (U/mg)	50.11±0.72	72.46±1.19	67.31±1.13
	K_m_(mM)	0.8751±0.03	0.65±0.03	0.5853±0.03
**ATP**	*k*_cat_ (s^-1^)	37.58	54.39	50.48
	*k*_cat_/K_m_ (mM^-1^s^-1^)	42.94	83.69	86.24

### PKA phosphorylation increases the sensitivity of CKβ to HC-3 inhibition

**Fig 7:**
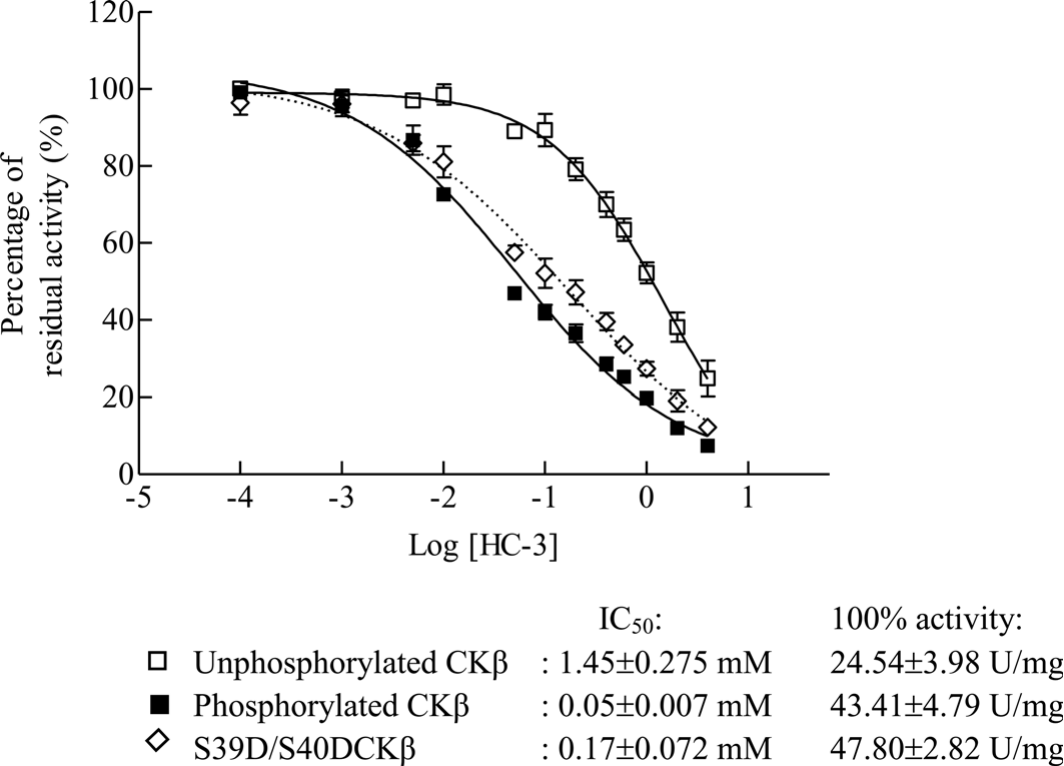
Effect of PKA phosphorylation and phosphorylation mimic mutation of CKβ on the sensitivity to HC-3 inhibition. The activities of unphosphorylated, *in vitro* phosphorylated, and S39D/S40D-mutant CKβ were measured by PK-LDH-dependent coupled-enzyme assays as described in the experimental procedures, with 4 mM choline as substrate and the indicated HC-3 inhibitor concentrations. Each bar represents the standard error of the mean (SEM) from three independent experiments.

Hemicholinium-3 (HC-3), a potent choline uptake inhibitor, also acts as a competitive inhibitor of CK; it blocks the catalytic activity of CK by competing for the choline binding site. HC-3 is more potent in inhibiting the activity of CKα than that of the CKβ isozyme [5]. The substrate preference of CK isozymes had been postulated as representing one of the distinctive features that can be influenced by differential inhibitory potency of HC-3 [5]. In this study, we have shown that PKA phosphorylation alters the substrate preference of CKβ, hence PKA phosphorylation is predicted to modulate the inhibitory effect of HC-3. The half-maximal inhibitory concentration (IC_50_) values were determined for the unphosphorylated, *in vitro* PKA-phosphorylated, and the S39D/S40D phosphorylation-mimic mutant CKβ. As shown in Fig 7, PKA phosphorylation rendered CKβ more sensitive to HC-3 inhibition. The half-maximal inhibitory concentration (IC_50_) of phosphorylated CKβ and of the phosphorylation mimic at saturating choline and ATP concentrations were decreased by 29- and 9-fold, respectively, as compared to unphosphorylated CKβ (Fig 7). The drastic IC_50_ decrease implies more efficient HC-3 inhibition of phosphorylated CKβ as compared to the unphosphorylated form of the enzyme.

## Discussion

CKβ is one of the CK isozymes involved in phospholipid biosynthesis. Although CKβ is the least active isoform with respect to choline and ethanolamine phosphorylation [5], it has a specific role for muscle development and bone homeostasis [6, 8, 11, 12] that cannot be replaced by CKα. Thus, characterization of CKβ post-translational modification by phosphorylation is relevant for better understanding of this functionally non-redundant protein. In this study, we have identified PKA as the protein kinase responsible for the *in vitro* and intracellular phosphorylation of CKβ. The level of CKβ phosphorylation is regulated by cAMP, the second messenger for PKA activation. The first indication that CKβ is regulated by PKA activity was based on the inhibition of choline incorporation into phosphatidylcholine by H-89, a small-molecule PKA inhibitor [29]. However, post-translational modification of choline kinase isozymes through phosphorylation by PKA was not reported. The in-gel kinase assay used in this study might not detect phosphorylation of CKβ by other kinases due to inefficient renaturation of kinases in the cell extract; also, some kinases are only active when bound to their essential cofactors. Therefore, the possibility of CKβ phosphorylation by other protein kinases cannot be excluded. We note that H-89 used as intracellular PKA inhibitor in this study has also been found to inhibit other protein kinases like Rho-associated kinase (ROCK), AMP-activated kinase (AMPK), mitogen- and stress-activated protein kinase (MSK1) and ribosomal protein S6 kinase (S6K1) [39]. However, the experiments utilizing H-89 were performed in combination with forskolin and IBMX treatment that increase the level of the PKA activator cAMP.

The observed phosphorylation of CKβ in the N-terminal region of the protein at residues Ser 39 and Ser 40 is very similar to PKA phosphorylation of yeast CK which was also found in the N-terminal domain residues Ser 30 and Ser 85 [26, 27]. The phosphorylated residues identified in both human CKβ and yeast CK are not located in the catalytic domains of the enzymes. In contrast to CKβ, phosphorylation of human CKα occurs at a tyrosine located in the choline-binding groove close to the sequence element termed Brenner’s motif, a characteristic of phosphoryltransfer enzymes [24]. Based on the global analysis of cyclin-dependent kinase 1 (Cdk1) substrate phosphorylation, more than 90% of phosphorylation sites were predicted to be located in disordered regions or loops of the proteins [40]. Phosphorylation in disordered or non-conserved regions was proposed as a nonspecific mechanism that could disrupt, or generate, interactions with other proteins [40, 41]. In the case of CKβ, it remains to be studied whether the effect of PKA phosphorylation on enzyme properties observed in this study is due to allosteric regulation or nonspecific mechanisms such as changes in electrostatic properties [41]. According to www.phosphosite.org, phosphorylation of CKβ Ser 39 and Ser 40 by Akt [42] and Aurora [43] kinases had also been identified by mass spectrometry. It is therefore possible that the effect of PKA-induced phosphorylation observed in this study can also be produced by these kinases.

PKA phosphorylation increased the catalytic activity of CKβ, similarly to human CKα and yeast CK [24, 26]. In yeast, PKA phosphorylation increased choline kinase activity by about 3-fold [26]. Tyrosine phosphorylation of human CKα increased its ATP:choline phosphoryltransfer activity by 1.4-fold, whereas co-expression with its interacting partner EGFR further stimulated its kinase activity about 2.5-fold [24]. Enhanced enzyme activity resulting from phosphorylation was shown to elevate the rate of cell proliferation. Treatment with epidermal growth factor (EGF) increased the proliferation of cells transfected with wild type CKα, but not of cells transfected with CKα devoid of PKA phosphorylation sites [24].

Previously, it was reported that CKβ particularly acted as ethanolamine kinase in the cell because overexpression of CKβ increased the level of only phosphoethanolamine, but not of phosphocholine [20]. In our study, the catalytic efficiency (indicated by *k*_*cat*_/K_m_) of PKA-phosphorylated CKβ, with choline as substrate, was significantly higher compared to the unphosphorylated enzyme, whereas the catalytic efficiency with ethanolamine as substrate was almost unchanged. Therefore, PKA phosphorylation might shift CKβ’s preferential activity from ethanolamine kinase to choline kinase, which could make CKβ become the main contributor of phosphocholine, a putative second messenger in the regulation of mitogenesis [44]. The alteration of the cellular phosphocholine level by CKβ renders this isoform, especially in tumors that do not overexpress CKα [45], a potential target in anticancer strategies targeting homeostasis of choline metabolism.

The S39D/S40D CKβ mutant produced in this study was able to mimic the effect of PKA phosphorylation as evidenced by the high similarities in catalytic properties of the phosphorylated enzyme and the aspartate mutant. Mimicking protein phosphorylation by replacing phosphorylated serine with an amino acid containing a negatively charged side chain like aspartate or glutamate has been used as a tool to study biochemical and cell-biological effects of protein phosphorylation [46, 47]. It was suggested that phosphorylation sites evolved from the acidic residues of glutamate or aspartate [48], and crystal structures of several proteins showed that both phosphorylated serine and acidic Asp or Glu residues formed the same electrostatic interactions with basic amino acids [48]. Cell transfection using the phosphorylation mimic produced in this work can serve to investigate the cellular consequences of phosphorylation especially in settings when the level of wild type CKβ phosphorylation cannot be easily controlled.

HC-3 is an inhibitor that suppresses the activity of CK [5] by competing with choline for the same binding site. Among CK isozymes, CKα is more sensitive to HC-3 than is CKβ due to a slight difference in the structure of the choline-binding groove. In this study, we have shown PKA phosphorylation to increase the sensitivity of CKβ to HC-3 inhibition. The IC_50_ value for the phosphorylated CKβ decreased by 29-fold compared to the unphosphorylated enzyme. In the case of competitive inhibition, the inhibitory constant, *K*_i_, can be calculated from IC_50_ values using the formula *K*_i_ = IC_50_/(S/K_m_+1) [49]. For our experimental setting, *K*_i_ for the unphosphorylated CKβ was calculated to be 194 μM HC-3, which is slightly higher than the value of 116 μM previously reported [5]. Following PKA-induced phosphorylation, the *K*_i_ of CKβ dropped drastically to 4.7 μM, and this value is very similar to the *K*_i_ determined for CKβ mutants exhibiting higher structural flexibility near the choline-binding groove [5]. This finding may indicate that PKA phosphorylation induces a conformational change that leads to higher flexibility at the CKβ choline-binding pocket to accommodate HC-3.

Compared with the lipid-dependent protein kinase C, very little involvement of PKA in phospholipid metabolism has been reported. This study highlights PKA phosphorylation of CKβ at serine residues 39 and 40, showing that cellular CKβ phosphorylation can be regulated by the level of cAMP. Phosphorylation of CKβ increased the catalytic efficiencies for the substrates choline and ATP, as well as its sensitivity to HC-3 inhibition. Substituting aspartate residues for particular serine phosphorylation sites in CKβ mimicked the effect of PKA phosphorylation on the enzyme’s catalytic properties. In mouse, the level of CKβ is relatively higher than that of CKα in brain, heart and liver tissues [2, 50]. Therefore, it is predicted that modulation of CKβ activity by PKA will have a more prominent effect on choline metabolism in human tissues with higher expression levels of CKβ. Whereas the CKα isozyme has become a target for interference with phospholipid metabolism in anticancer strategies, the pronounced alteration of CKβ catalytic activity and inhibition by HC-3 observed after PKA phosphorylation strongly suggests that this isoform could also be implicated in carcinogenesis and be considered as potential target for anti-choline kinase drugs, especially in liver and thymus tumors that do not overexpress CKα [45]. Decreased phosphatidylcholine in CKβ knockout mice impaired mitochondrial function and muscle regenerative capacity [6]. Thus, it is likely that PKA stimulation of CKβ towards the synthesis of phosphatidylcholine in muscle cells is crucial for mitochondrial membrane stability and muscle generation. PKA phosphorylation of CKβ that leads to higher levels of phosphocholine could also promote bone mineralization since reduced phosphocholine levels in CKβ-deficient osteoblasts resulted in depleted inorganic phosphate essential for bone mineralization [11, 12]. In conclusion, this study has demonstrated significant PKA-dependent regulation of CKβ’s biochemical properties that may affect cellular choline metabolism and cell growth. Future work will aim to investigate the phosphorylation state of CKβ in normal and in cancer cells as well as the effect of phosphorylation on CKβ interaction with CKα. It will be interesting to determine which of the serines (39 or 40) is more critical for the effect of PKA phosphorylation *in vitro* and *in vivo*, and whether S39D or S40D single mutants can mimic the effect.

## Materials and Methods

### Expression and purification of wild type and mutant CK proteins

Wild type and mutant CKβ, CKα, and yeast CK proteins were expressed in *Escherichia coli* BL21(DE3) using plasmids pET-14b (Novagen) for producing N-terminal His_6_-tagged proteins, or pGEX-RB [51] for GST-fusion proteins (Table 3). Recombinant proteins were produced as described previously [45]. Protein concentration was determined by the Bradford assay (Bio-Rad).

**Table 3:** Proteins and expression plasmids used in this study.

Proteins	Plasmids	Reference
CKα2	pET-14bCKα2	[45]
yCK	pGEX-RByCK	this study
CKβ	pET-14bCKβ	[21]
	pGEX-RBCKβ	[51]
	pEGFP-C1-*Nde*ICKβ	this study
Truncation at 42 amino acid	pGEX-RB∆42NCKβ	this study
Single mutant CKβ^S39A^	pGEX-RBS39ACKβ	this study
Single mutant CKβ^S40A^	pGEX-RBS40ACKβ	this study
Single mutant CKβ^S42A^	pGEX-RBS42ACKβ	this study
Double mutant CKβ^S39AS40A^	pGEX-RBS39AS40ACKβ	this study
	pEGFP-C1-*Nde*IS39AS40ACKβ	this study
Double mutant CKβ^S39AS42A^	pGEX-RBS39AS42ACKβ	this study
Double mutant CKβ^S40AS40A^	pGEX-RBS40AS42ACKβ	this study

### Preparation of cell lysates

Lysates from MCF-7, HepG2, and HCT-116 cells for in-gel kinase and *in vitro* phosphorylation assays were prepared by lysing 5×10^6^ cells with 200 μL of Triton X-100 lysis buffer (0.1 M KH_2_PO_4_, pH 7.5, 0.1 mM EDTA, 0.1 mM EGTA, 10% (v/v) glycerol, 0.5% (v/v) Triton X-100). The cells were lysed on ice for 30 minutes. Cell debris was removed by centrifugation at 16,000× g, at 4°C for 10 minutes.

### In-gel kinase assay

In-gel kinase assays were performed as described by Wooten et al. [52]. Two milligrams of the CKβ protein substrate were included in the gels. For negative control, the same amount of bovine serum albumin was included. Sixty micrograms of the cell lysates were boiled for 5 minutes at 95°C in 5× SDS loading buffer. Samples were separated by electrophoresis at 25 mA until the blue dye reached the bottom of the gel. The gel was then washed in 50 mL of SDS-removing buffer I (50 mM Tris-HCl, pH 8, 20% (v/v) isopropanol) for 1 hour, followed by washing three times with 50 mL of SDS-removing buffer II (50 mM Tris-HCl, pH 8, 1 mM β-mercaptoethanol) for 1 hour, and then the gel was incubated in 50 mL of denaturing buffer (50 mM Tris-HCl, pH 8, 6 M guanidine-HCl, 20 mM β-mercaptoethanol) for 30 minutes with gentle agitation. Traces of guanidine-HCl were later washed off twice with 50 mL of renaturation buffer [50 mM Tris-HCl, pH 8, 100 mM NaCl, 5 mM MgCl_2_, 5 mM β-mercaptoethanol, 0.04% (v/v) Tween-20] for 40 minutes. The solution was replaced after every 10 minutes. Subsequently, the gel was incubated in 50 mL of renaturation buffer for another 3 hours at 4°C without agitation. The solution was replaced, and the incubation was continued for 16 hours with the same conditions.

Prior to the kinase reaction, the gel was washed twice with 50 mL of kinase buffer (40 mM HEPES, pH 7.6, 10 mM MgCl_2_, 2 mM β-mercaptoethanol, 0.1 mM EGTA) for 1 hour with gentle agitation. For the kinase reaction, the gel was incubated with 50 μM of 5 μCi/mL [γ^_32^P]ATP in 3 mL of kinase buffer for 4 hours with gentle agitation. For inhibition assays, 15 μg/mL of PKI (Promega) were included in the kinase buffer. The reaction was terminated by washing the gel five times with 50 mL of stop buffer [5% (w/v) trichloroacetic acid; 1% (w/v) sodium pyrophosphate] for 75 minutes. The gel was dried on cellophane membrane for 16 hours at room temperature, and finally the radioactive signal was detected using X-ray film (Kodak).

### *In vitro* phosphorylation and inhibition assays

*In vitro* phosphorylation of CKβ was performed by incubating 20 μg of CKβ protein with 80 U of PKA catalytic subunit (Promega) or 20 μg of cell lysate in 50 mM KH_2_PO_4_, pH 7.5, 0.1 mM EDTA, 0.1 mM EGTA, 20 mM MgCl_2_, 10 mM β-mercaptoethanol, 3 mM of [γ^_32^P]ATP (1 μCi) or unlabeled ATP, 1× complete protease (Roche) and 1× phosphatase inhibitor cocktail (Calbiochem) in a total volume of 40 μL at 30°C for 1 hour. The reaction was terminated by heating at 95°C for 5 minutes in 2× SDS loading buffer. *In vitro* phosphorylation of other human and yeast CKs was performed as described above using 80 U of PKA. Samples containing 5 or 10 μg of phosphorylated protein per well were analyzed by 10% SDS-PAGE. The phosphoprotein was detected by either autoradiography or Pro-Q Diamond phosphoprotein gel stain (Invitrogen). The phosphoprotein gel staining was performed according to the manufacturer’s protocol. The level of protein loading was determined by Coomassie blue stain or SYPRO^®^ Ruby stain (Invitrogen). For inhibition assays, 15 to 45 μg/mL of PKI (Promega) were added to the reaction mixture. To investigate the effect of PKA and ATP concentrations on the level of CKβ phosphorylation, 20 to 120 U of PKA and 25 to 100 μM of ATP were used in the *in vitro* CKβ phosphorylation reactions.

### Identification of PKA phosphorylation sites

PKA phosphorylation sites were identified by mass spectrometry and mutagenesis. Phosphorylated samples were run on 12% SDS-PAGE gel, the gels were stained with Coomassie Brilliant Blue, the protein bands were excised from the gels and analyzed by the Bioanalytical Mass Spectrometry Service Unit (Max Planck Institute for Biophysical Chemistry, Goettingen, Germany) to determine the phosphorylation sites using the TiO_2_ chromatography procedure for phosphopeptide enrichment [53]. Mass spectrometry analysis was performed on the *in vitro*-phosphorylated purified CKβ protein to determine PKA phosphorylation sites using the Mascot Search Program (Matrix Science, USA). Data analysis was independently repeated using MaxQuant [54] and Proteome Discoverer [55] to reconfirm previously identified phosphorylation sites.

The PKA phosphorylation sites were subsequently studied by mutagenesis. Serine-to-alanine (phosphorylation-negative alteration) or serine-to-aspartate (phosphorylation-mimic) mutations of CKβ were created by one-step PCR mutagenesis utilizing the unique *Asc*I recognition site at nucleotides 107–114 of the CKβ open reading frame. The mutant DNA sequences were amplified by using the forward mutagenesis primers listed in Table 4 and CKβ-*BamH*I-3’ reverse primer. The amplified products were digested with *Asc*I and *BamH*I restriction enzymes, purified, and then ligated in the pET-14bCKβ vector digested with the same restriction enzymes. For generating the ∆42N-CKβ N-terminal truncation mutant to show that PKA phosphorylation was confined to the first 42 amino acids of CKβ, the gene fragment was amplified by using the corresponding CKβ-*Nde*I-5’ forward primer (Table 4) and the CKβ-*BamH*I-3’ reverse primer. The PCR product was digested with *Nde*I and *BamH*I and cloned into pGEX-RB. All plasmid constructs were verified by DNA sequencing.

**Table 4:** Oligonucleotides used in this study.

Oligonucleotides	Sequence (5’ to 3’)
42N truncation CKβ-*Nde*I-5’	GAATTCCATATGCGTGACGCCGAGCGCC
S39ACKβ-*Asc*I-5’	AAAACGGCGGCGCGCCGCGTCGCTGTCGCGTG
S40ACKβ-*Asc*I-5’	AAAACGCCGGCGCGCCTCGGCGCTGTCGCGTGACG
S42ACKβ-*Asc*I-5’	AAAACGGCGGCGCGCCTCGTCGCTGGCGCGTGACGCCG
S39AS42ACKβ-*Asc*I-5’	AAAACGGCGGCGCGCCGCGTCGCTGGCGCGTGACGCCG
S40AS42ACKβ-*Asc*I-5’	AAAACGGCGGCGCGCCTCGGCGCTGGCGCGTGACGCCG
S39AS40ACKβ-*Asc*I-5’	AAAACGGCGGCGCGCCGCGGCGCTGTCGCGTGACG
S39DS40DCKβ-*Asc*I-5’	AAAACGGCGGCGCGCCGATGATCTGTCGCGTGACG
CKβ-*BamH*I-3’	CGCGGATCCTCAGGATGAGGAGTGGACACTGG

### Cell cultures and establishment of a stable CKβ-expressing cell line

Cells were maintained in complete DMEM (Invitrogen) supplemented with 10% fetal bovine serum (FBS) at 37°C, 95% humidity and 5% CO_2_. Stable transfection of pEGFP-C1-*Nde*ICKβ for the expression of N-terminally GFP tagged CKβ was performed in the HEK293 cell line. The pEGFP-C1-*Nde*I vector was modified from the pEGFP-C1 vector (Clontech) by first deleting the existing *Nde*I site at positions 233–238 by T4 DNA polymerase filling after restriction enzyme cleavage, followed by introduction of a short linker (5’-CAAGCTTCA**CATATG**GCTGGTACC-3’) containing a new *Nde*I site (bold faced) between the *Hind*III and *Kpn*I sites in the multiple cloning site of the original vector. Cells were transfected by using Lipofectamine™ 2000 (Invitrogen) according to the manufacturer’s recommendations. Approximately 10^5^ cells were seeded into 24- well plates a day before the transfection. Plasmid (500 ng) was diluted with 25 μL of Opti-MEM (Invitrogen) and mixed with an equal volume of diluted Lipofectamine (0.5 μL of Lipofectamine in 25 μL of Opti-MEM). The mixture was incubated at room temperature for 15 minutes before being added to the cells. The medium containing the transfection reagent was replaced after 6 hours. At 48 hours post transfection, cells were split with 1:10 dilutions and grown in medium containing 1 mg/mL of geneticin antibiotic (G418, from Sigma). The medium was replaced every three days until there was no massive cell death and the cells reached confluence. The cells were grown for another two passages in medium containing 1 mg/mL of G418. Expression of EGFP-CKβ in stably transfected cells was confirmed one month post transfection using anti-GFP antibody (Abcam).

### Treatment with forskolin, IBMX, and H-89

Cells (1.5×10^5^) were seeded in 24-well plates and allowed to grow for 24 hours before serum starvation for another 24 hours by replacing the complete medium with medium containing 0.5% FBS. Cells were treated with 20 μM of forskolin (adenylyl cyclase-activating diterpene) and 100 μM of IBMX (3-isobutyl-1-methylxanthine; phosphodiesterase inhibitor) for 15–60 minutes. For experiments with H-89 (small-molecule PKA inhibitor), the cells were pretreated with 20 μM of H-89 for 1 hour.

### Immunoprecipitation and Western blot detection

Cells were harvested and lysed with ProteoJET™ mammalian cell lysis reagent (Thermo Scientific). The lysates were diluted ten times with NP (nonyl phenoxypolyethoxylethanol) lysis buffer [25 mM HEPES, pH 7.4, 2.5 mM EDTA, 50 mM NaCl, 50 mM NaF, 30 mM sodium pyrophosphate, 10% (v/v) glycerol, 10% (v/v) NP-40]. GFP-CKβ protein was immunoprecipitated with CKβ antibody [21]: Here, 0.5 μg of CKβ antibody was added to 200 μL of the diluted cell lysate and incubated at 4°C for 16 hours. After that, 10 μL of protein G plus/protein A-agarose suspension (Thermo Scientific) were added and incubated for another 2 hours to capture the antigen-antibody complexes. The agarose suspension was then washed with NP lysis buffer to remove unbound protein, and finally the immunoprecipitate was eluted with 20 μL of elution buffer containing glycine-HCl (pH 2.5). The eluted samples were boiled in 4 μL of 5 × loading buffer at 95°C for 5 minutes before being loaded for SDS-PAGE followed by Western transfer to nitrocellulose membrane.

The phosphoprotein was detected by phospho-(Ser/Thr) PKA substrate (PhosphoPKAS) antibody (Cell Signaling Technology) diluted at 1:1000 ratio with blocking buffer (Tris-buffered saline containing 5% BSA). The primary antibody was incubated with the membrane at 4°C for 16 hours followed by detection with rabbit IgG-horseradish peroxidase (HRP)-conjugated secondary antibody (1: 5,000 dilution). The signal was developed by Supersignal West Pico chemiluminescent substrate (Thermo Scientific) and detected by using the FUSION-FX chemiluminescence documentation system (Vilber Lourmat). After imaging, the antibody bound on the membrane was removed with stripping buffer [0.2 M glycine, pH 2.5, 0.1% (w/v) SDS, 1% (v/v) Tween-20], and the membrane was reprobed with rabbit anti-GFP antibody (Abcam) at 1:1000 dilution.

For the detection of CKβ-phosphorylating kinase in the in-gel kinase assay, a replicated gel was blotted onto nitrocellulose membrane followed by Western detection with 1:5,000 dilution of PKA catalytic subunit-recognizing polyclonal antibody (Abcam) according to similar procedures as described above.

### *In vitro* assay of choline kinase

Choline kinase activity was determined by using the pyruvate kinase (PK)-lactate dehydrogenase (LDH) coupled-enzyme assay as previously described [56] using a modified buffer. The reaction mixture contained 100 mM HEPES, pH 8.5, 100 mM KCl, 10 mM MgCl_2_, 0.5 mM phosphoenolpyruvate, 0.25 mM NADH, 2 mM ATP, 4 U of pyruvate kinase, 5 U of lactate dehydrogenase, 1 to 2 μg of purified CK protein and 4 mM choline chloride or 4 mM ethanolamine in a final volume of 1 mL. The reaction was performed at 37°C and initiated by adding choline or ethanolamine. Any background activity before the addition of choline or ethanolamine was subtracted from the total activity. The purified CK protein used in such assays was either unphosphorylated, *in vitro* phosphorylated (as described above), or the S39D/S40D phosphorylation mimic mutant. The *in vitro* phosphorylated CKβ was used immediately for enzymatic assay after the 1 hour PKA treatment. The unphosphorylated CKβ wild type and the S39D/S40D CKβ variant were either freshly purified proteins or purified proteins stored at −80°C. All the kinetic parameters were obtained from nonlinear regression curve fitting of the Michaelis-Menten plot by using GraphPad Prism version 5.0.

### Hemicholinium-3 inhibition of CKβ

The effect of PKA-induced phosphorylation on HC-3 inhibition of CKβ was investigated by comparing the half-maximal inhibitory concentration (IC_50_) values of the unphosphorylated, PKA- phosphorylated, and the S39D/S40D phosphorylation-mimic mutant CKβ. IC_50_ was determined by the standard CK assay as described above in the presence of saturating substrate concentration (4 mM choline) and increasing HC-3 concentrations, and fitting the data plotted in a dose-response curve by using GraphPad Prism 5.0.

## Supporting Information

S1 Fig Mass spectrometry analysis of phosphorylated CKβ. (PDF)

S2 Fig Effect of the phosphorylation mimic double-mutation on the catalytic activity of CKβ with choline as substrate, at constant ATP concentration. (PDF)

S3 Fig Effect of the phosphorylation mimic double-mutation on the catalytic activity of CKβ with ethanolamine as substrate, at constant ATP concentration. (PDF)

S4 Fig Effect of the phosphorylation mimic double-mutation on the catalytic activity of CKβ with ATP as substrate, at constant choline concentration. (PDF)
