# Supplementary material for: Phosphorylation of Human Choline Kinase Beta by Protein Kinase A: Its Impact on Activity and Inhibition
Source: PLoS One. 2016 May 5;11(5):e0154702. doi: 10.1371/journal.pone.0154702 (PMC4858151; doi:10.1371/journal.pone.0154702)
Supplement: S1 Fig — (PDF) [file pone.0154702.s001.pdf]

Protein Family Summary

Filter | Significance threshold p< 0.05 | Max. number of families 2000 | [help]
Ions score or expect cut-off 0 | Dendrograms cut at 0

Proteins (8) | Quantitation (8) | Unassigned (1320)

Protein families 1–8 (out of 8)

10 per page 1 Expand all Collapse all

Accession Find Match case

▼1 gi|6978649 284 choline/ethanolamine kinase [Homo sapiens]

		Score	Mass	Matches	Sequences	emPAI	
1.1	gi|6978649	284	45242	37 (13)	22 (11)	1.50	choline/ethanolamine kinase [Homo sapiens]

▶1 sameset of gi|6978649

▼37 peptide matches (36 non-duplicate, 1 duplicate)

☑ Auto-fit to window

Query	Dupes	Observed	Mr(expt)	Mr(calc)	ppm	M	Score	Expect	Rank	U	Peptide
67		388.2009	774.3872	774.3872	0.092	0	18	0.86	▶1	U	K.DGLQQSK.C
103		416.2225	830.4304	830.4286	2.20	0	22	0.17	▶1	U	R.HYLAEAK.K
125		435.7375	869.4604	869.4607	-0.25	0	1	4.1	▶3	U	R.VQPEELR.V
168	▶1	468.6884	935.3622	935.3627	-0.45	1	27	0.11	▶1	U	R.RASSLSR.D + 2 Phospho (ST)
241		**501.7458**	**1001.4770**	**1001.4778**	**-0.75**	**0**	**36**	**0.0022**	▶1	**U**	**K.GQLTSVHSSS.-**
248		506.7555	1011.4964	1011.4975	-0.99	2	15	1.3	▶2	U	R.RRASSLSR.D + Phospho (ST)
265		513.7877	1025.5608	1025.5618	-0.90	1	21	0.42	▶1	U	R.RVQPEELR.V
327		**541.7293**	**1081.4440**	**1081.4441**	**-0.068**	**0**	**38**	**0.00096**	▶1	**U**	**K.GQLTSVHSSS.- + Phospho (ST)**
348		546.7375	1091.4604	1091.4638	-3.05	2	4	1.2	▶4	U	R.RRASSLSR.D + 2 Phospho (ST)
350		546.7388	1091.4630	1091.4638	-0.67	2	7	1.1	▶2	U	R.RRASSLSR.D + 2 Phospho (ST)
351		364.8284	1091.4634	1091.4638	-0.37	2	16	1.3	▶2	U	R.RRASSLSR.D + 2 Phospho (ST)
360		550.3219	1098.6292	1098.6284	0.73	0	12	0.71	▶1	U	R.EPVLSAAIATK.M
408		568.2827	1134.5508	1134.5498	0.89	0	17	1.1	▶2	U	R.FQFYFQQK.G
478		586.2582	1170.5018	1170.5030	-0.98	1	15	1.3	▶2	U	R.ASSLSRDAER.R + Phospho (ST)
487		**588.7774**	**1175.5402**	**1175.5418**	**-1.34**	**0**	**56**	**0.00017**	▶1	**U**	**K.GETLSQEEQR.K**
591		626.2416	1250.4686	1250.4693	-0.53	1	18	0.19	▶1	U	R.ASSLSRDAER.R + 2 Phospho (ST)
592		626.2424	1250.4702	1250.4693	0.75	1	0	1	▶1	U	R.ASSLSRDAER.R + 2 Phospho (ST)
668		**652.8253**	**1303.6360**	**1303.6368**	**-0.56**	**1**	**70**	**1.3e-005**	▶1	**U**	**K.KGETLSQEEQR.K**
669		435.5532	1303.6378	1303.6368	0.76	1	24	0.057	▶1	U	K.KGETLSQEEQR.K
721		448.5933	1342.7581	1342.7608	-2.07	0	9	1.2	▶2	U	R.LEQYIPSRPLK.T
722		**672.3873**	**1342.7600**	**1342.7608**	**-0.60**	**0**	**36**	**0.019**	▶1	**U**	**R.LEQYIPSRPLK.T**
788		704.2911	1406.5676	1406.5704	-1.97	2	10	2.4	▶3	U	R.RASSLSRDAER.R + 2 Phospho (ST)
789		704.2917	1406.5688	1406.5704	-1.12	2	11	1.2	▶5	U	R.ASSLSRDAERR.A + 2 Phospho (ST)
790		469.8636	1406.5690	1406.5704	-1.03	2	9	1.2	▶3	U	R.RASSLSRDAER.R + 2 Phospho (ST)
792		704.2921	1406.5696	1406.5704	-0.55	2	15	0.29	▶1	U	R.RASSLSRDAER.R + 2 Phospho (ST)
811		**715.3977**	**1428.7808**	**1428.7824**	**-1.07**	**1**	**67**	**8.6e-006**	▶1	**U**	**R.KLEEDLLVEVSR.Y**
867		744.2761	1486.5376	1486.5367	0.61	2	5	0.82	▶1	U	R.RASSLSRDAER.R + 3 Phospho (ST)
900		**760.3979**	**1518.7812**	**1518.7831**	**-1.21**	**0**	**49**	**0.00013**	▶1	**U**	**R.SLGPQLYGVFPEGR.L**
906		**761.4261**	**1520.8376**	**1520.8351**	**1.67**	**0**	**41**	**0.00033**	▶1	**U**	**R.VYPVSGGLSNLLFR.C**
976		534.8962	1601.6668	1601.6677	-0.56	0	20	0.4	▶1	U	K.MAQFHGMEMPFTK.E + 3 Oxidation (M)
977		**801.8425**	**1601.6704**	**1601.6677**	**1.74**	**0**	**40**	**0.0018**	▶1	**U**	**K.MAQFHGMEMPFTK.E + 3 Oxidation (M)**
1012		**827.8621**	**1653.7096**	**1653.7070**	**1.63**	**1**	**30**	**0.012**	▶1	**U**	**K.DGLQQSKCPDTTPK.R + Carbamidomethyl (C); Phospho (ST)**
1058		**564.9383**	**1691.7931**	**1691.7937**	**-0.38**	**0**	**31**	**0.0032**	▶1	**U**	**R.CSLPDHLPSVGEEPR.E + Carbamidomethyl (C)**
1059		**846.9056**	**1691.7966**	**1691.7937**	**1.73**	**0**	**34**	**0.003**	▶1	**U**	**R.CSLPDHLPSVGEEPR.E + Carbamidomethyl (C)**
1084		**863.9865**	**1725.9584**	**1725.9625**	**-2.33**	**1**	**46**	**6.9e-005**	▶1	**U**	**K.TQELREPVLSAAIATK.M**
1085		576.3269	1725.9589	1725.9625	-2.08	1	18	0.16	▶1	U	K.TQELREPVLSAAIATK.M

S1 Fig. Mass spectrometry analysis of phosphorylated CKβ. Fifteen peptide fragments were found to be phosphorylated; the range of the ion score was from 4 to 38. A total of 13 peptide fragments covered the amino acid sequence Arg 36 to Arg 43 (RRASSLSR).
