# Supplementary material for: Phosphorylation of Human Choline Kinase Beta by Protein Kinase A: Its Impact on Activity and Inhibition
Source: PLoS One. 2016 May 5;11(5):e0154702. doi: 10.1371/journal.pone.0154702 (PMC4858151; doi:10.1371/journal.pone.0154702)
Supplement: S2 Fig — (PDF) [file pone.0154702.s002.pdf]

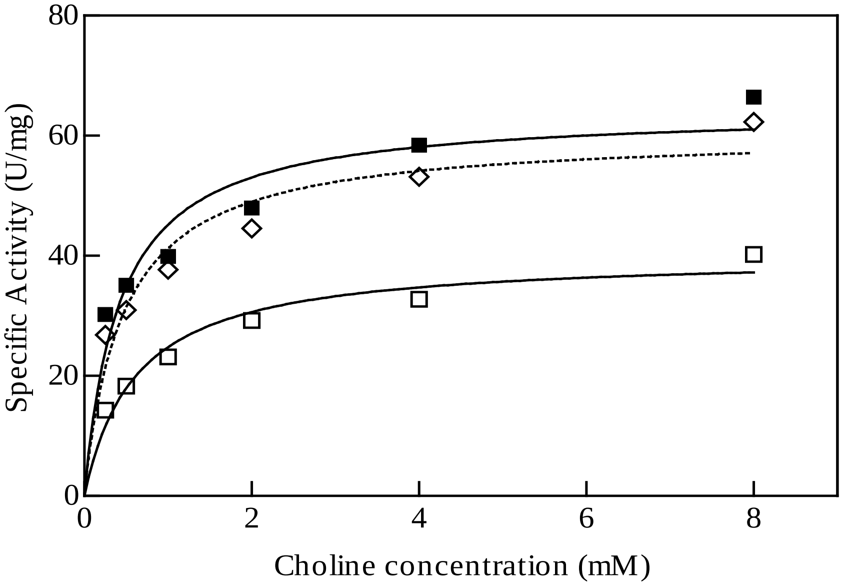

Unphosphorylated CKβ V_{max} : 40.14 ± 1.26 U/mg
K_m : 0.62 ± 0.072 mM

Phosphorylated CKβ V_{max} : 64.27 ± 2.39 U/mg
K_m : 0.42 ± 0.065 mM

S39D/S40D CKβ V_{max} : 60.42 ± 2.14 U/mg
K_m : 0.46 ± 0.067 mM

S2 Fig. Effect of the phosphorylation mimic double-mutation on the catalytic activity of CKβ with choline as substrate, at constant ATP concentration (2 mM). Each data point represents the average of three independent measurements.
