# Supplementary material for: Phosphorylation of Human Choline Kinase Beta by Protein Kinase A: Its Impact on Activity and Inhibition
Source: PLoS One. 2016 May 5;11(5):e0154702. doi: 10.1371/journal.pone.0154702 (PMC4858151; doi:10.1371/journal.pone.0154702)
Supplement: S3 Fig — (PDF) [file pone.0154702.s003.pdf]

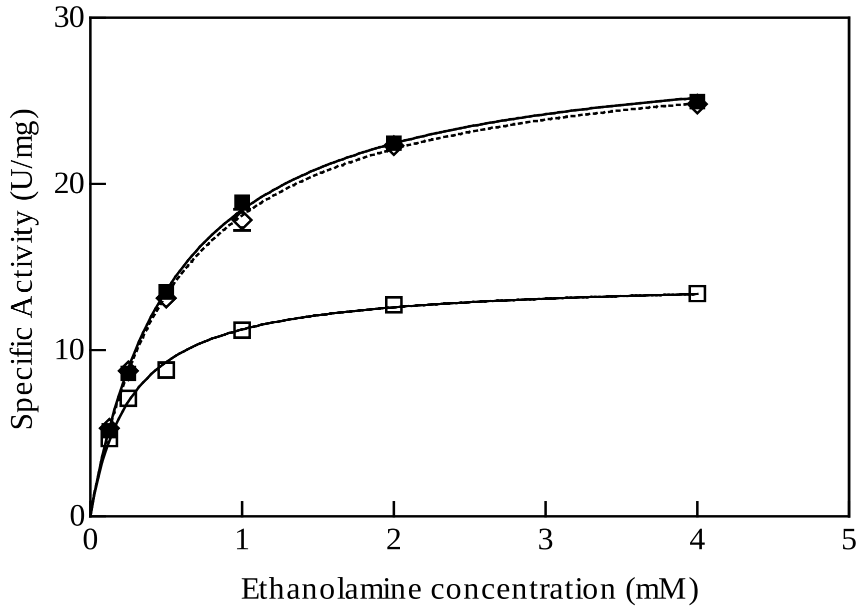

Unphosphorylated CKβ	V_{max}	: 14.28 ± 0.21 U/mg
	K_m	: 0.26 ± 0.015 mM
Phosphorylated CKβ	V_{max}	: 28.69 ± 0.28 U/mg
	K_m	: 0.55 ± 0.016 mM
S39D/S40D CKβ	V_{max}	: 28.39 ± 0.40 U/mg
	K_m	: 0.57 ± 0.024 mM

S3 Fig. Effect of the phosphorylation mimic double-mutation on the catalytic activity of CKβ with ethanolamine as substrate, at constant ATP concentration (2 mM). Each data point represents the average of three independent measurements.
