# Supplementary material for: Phosphorylation of Human Choline Kinase Beta by Protein Kinase A: Its Impact on Activity and Inhibition
Source: PLoS One. 2016 May 5;11(5):e0154702. doi: 10.1371/journal.pone.0154702 (PMC4858151; doi:10.1371/journal.pone.0154702)
Supplement: S4 Fig — (PDF) [file pone.0154702.s004.pdf]

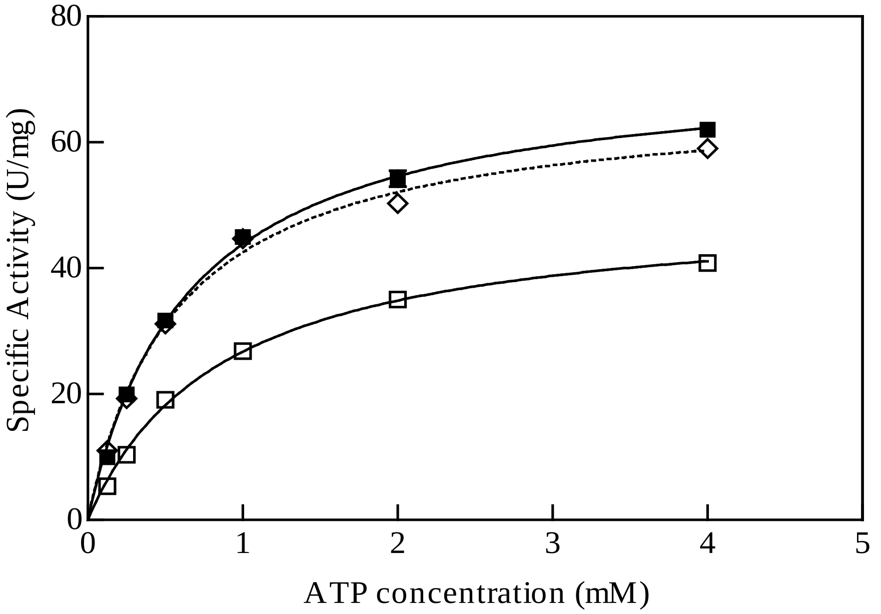

Unphosphorylated CKβ	V_{max}	: 50.11 ±0.72 U/mg
	K_m	: 0.87 ±0.034 mM
Phosphorylated CKβ	V_{max}	: 72.46 ± 1.19 U/mg
	K_m	: 0.65 ±0.032 mM
S39D/S40D CKβ	V_{max}	: 67.31 ± 1.13 U/mg
	K_m	: 0.58 ±0.030 mM

S4 Fig. Effect of the phosphorylation mimic double-mutation on the catalytic activity of CKβ with ATP as substrate, at constant choline concentration (4 mM). Each data point represents the average of three independent measurements.
